# ER stress affects processing of MHC class I-associated peptides

**DOI:** 10.1186/1471-2172-10-10

**Published:** 2009-02-16

**Authors:** Diana P Granados, Pierre-Luc Tanguay, Marie-Pierre Hardy, Étienne Caron, Danielle de Verteuil, Sylvain Meloche, Claude Perreault

**Affiliations:** 1 Department of Medicine, Institute for Research in Immunology and Cancer, Université de Montréal, Montréal, Canada; 2 Department of Pharmacology, Institute for Research in Immunology and Cancer, Université de Montréal, Montréal, Canada

## Abstract

**Background:**

Viral infection and neoplastic transformation trigger endoplasmic reticulum (ER) stress. Thus, a large proportion of the cells that must be recognized by the immune system are stressed cells. Cells respond to ER stress by launching the unfolded protein response (UPR). The UPR regulates the two key processes that control major histocompatibility complex class I (MHC I)-peptide presentation: protein synthesis and degradation. We therefore asked whether and how the UPR impinges on MHC I-peptide presentation.

**Results:**

We evaluated the impact of the UPR on global MHC I expression and on presentation of the H2K^b^-associated SIINFEKL peptide. EL4 cells stably transfected with vectors coding hen egg lysozyme (HEL)-SIINFEKL protein variants were stressed with palmitate or exposed to glucose deprivation. UPR decreased surface expression of MHC I but did not affect MHC I mRNA level nor the total amount of intracellular MHC I proteins. Impaired MHC I-peptide presentation was due mainly to reduced supply of peptides owing to an inhibition of overall protein synthesis. Consequently, generation of H2K^b^-SIINFEKL complexes was curtailed during ER stress, illustrating how generation of MHC I peptide ligands is tightly coupled to ongoing protein synthesis. Notably, the UPR-induced decline of MHC I-peptide presentation was more severe when the protein source of peptides was localized in the cytosol than in the ER. This difference was not due to changes in the translation rates of the precursor proteins but to increased stability of the cytosolic protein during ER stress.

**Conclusion:**

Our results demonstrate that ER stress impairs MHC I-peptide presentation, and that it differentially regulates expression of ER- vs. cytosol-derived peptides. Furthermore, this work illustrates how ER stress, a typical feature of infected and malignant cells, can impinge on cues for adaptive immune recognition.

## Background

The ultimate role of the immune system in host defense is to eliminate infected and transformed cells [1,2]. A fundamental feature of infected and neoplastic cells is that they are stressed cells [3-5]. In line with this, the innate immune system uses receptors such as NKG2D to recognize stressed cells [4,6,7]. One key question, however, is whether cellular stress can influence recognition of transformed or infected cells by the adaptive immune system [4,8].

The single feature uniting different stress stimuli (heat shock, hypoxia, viral replication, abnormal proteins, starvation or transformation) is that they all ultimately lead to accumulation of unfolded or misfolded proteins in the lumen of the ER [4,5]. Infection and neoplastic transformation increase protein translation and thereby the folding demand on the ER [9,10]. This is particularly true for cells submitted to hypoxia, nutrient deprivation or low pH in poorly vascularized bulky tumors, metastases and sites of inflammation [11,12]. Moreover, acquisition of numerous mutations during tumor progression leads to accumulation of abnormal proteins with an increased propensity to misfolding that further raises the ER folding burden [3,13].

The ER responds to the accumulation of unfolded proteins by activating intracellular signal transduction pathways, collectively called the unfolded protein response (UPR) [14,15]. The UPR is a highly conserved adaptive response that allows survival to limited stress but leads to apoptosis in the presence of overwhelming stress [16,17]. Mammalian UPR acts through three main transducers (PERK, ATF6 and IRE1) that are activated by dissociation of the master chaperone BiP/GRP78 [5,15]. Activation of PERK leads to phosphorylation of the translation initiation factor eIF2α and attenuation of cap-dependent translation [18]. The endonuclease activity of IRE1 generates a frameshift splice variant of XBP-1 encoding an active transcription factor that activates genes involved in protein degradation and controls the transcription of chaperones [19-21]. Targets of the cleaved active form of ATF6 include the chaperones BiP and GRP94, and the transcription factors XBP-1 and CHOP [17,19]. Activation of these UPR transducers has pervasive effects on cellular protein economy: i) attenuation of protein translation, ii) increased degradation of ER proteins by ER-associated degradation (ERAD), iii) transcriptional activation of genes involved in the folding machinery of the ER and iv) increased degradation of ER-localized mRNAs [14,22].

Presentation of MHC I-associated peptides to CD8 T cells is tightly linked to protein economy. MHC I peptides are preferentially generated from newly synthesized but rapidly degraded polypeptides relative to slowly degraded proteins [23,24]. Following proteasomal degradation, peptides are translocated into the ER where they undergo N-terminal trimming, loading onto MHC I/β_2_-microglobulin (β2m) heterodimers and export at the cell surface [25-29]. Since the UPR regulates the two key processes that shape MHC I peptide processing (protein translation and degradation) we reasoned that ER stress should impinge on MHC I peptide presentation. We addressed this question and found that MHC I presentation was impaired during ER stress induced by palmitate or glucose starvation. Moreover, ER stress differentially affected presentation of peptides derived from a protein localized in the ER vs. the cytosol.

## Results

### Engineering of K^b^-SIINFEKL stable transfectant cell lines

**Figure 1:**
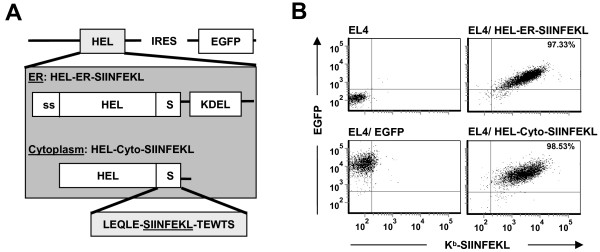
**EL4 stable transfectants express the SIINFEKL peptide derived from HEL targeted to the ER or to the cytosol**. (A) Schematic representation of the constructs used to generate EL4 stable transfectants. Modified coding sequences of HEL [34,35] in frame with the region coding for the ovalbumin-derived peptide SIINFEKL and its flanking region were cloned into the pIRES-EGFP2 vector. HEL-ER-SIINFEKL possesses HEL N-terminal signal sequence (ss) and the ER-retention signal KDEL and targets HEL to the ER; HEL-Cyto-SIINFEKL lacks the N-terminal signal sequence and the ER-retention signal and targets HEL to the cytoplasm (see materials and methods). (B) EL4 stable transfectants express K^b^-SIINFEKL at the cell surface. EL4 cells were transfected with the pIRES-EGFP2 vector encoding HEL-ER-SIINFEKL or HEL-Cyto-SIINFEKL. Stable transfectants were selected by repeated cycles of FACS of EGFP-positive cells combined with drug resistance (1000 μg/ml of G418). Cells were stained with 25-D1.16 monoclonal antibody, recognizing the K^b^-SIINFEKL complex, followed by staining with APC-conjugated anti-mouse IgG_1 _as secondary antibody. Depicted in the graphs are EGFP and K^b^-SIINFEKL MFI values of untransfected EL4 cells (*upper left*), EGFP-transfected cells (*lower left*) and the two representative clones that were used in further studies: EL4/HEL-ER-SIINFEKL (*upper right*) and EL4/HEL-Cyto-SIINFEKL (*lower right*). Percentages represent the proportion of cells expressing EGFP and K^b^-SIINFEKL.

Evidence suggests that subcellular localization of a protein (e.g., in the cytosol vs. the secretory pathway) may influence MHC I presentation of peptides derived from that specific protein [30-32]. Moreover, the UPR is primarily orchestrated to decrease protein overload in the ER [14,15]. We therefore wished to determine whether the UPR would differentially affect MHC I presentation of peptides derived from a precursor protein located in the cytosol versus the ER. To this end, we created stable EL4 transfectant cell lines expressing a chimeric protein located either in the ER or the cytoplasm (Figure 1A). We selected the EL4 thymoma cell line as a model because it expresses relatively high levels of MHC I [33] which allows us to assess changes of MHC I abundance over a wide dynamic range. To create the chimeric constructs, a minigene coding for the SIINFEKL peptide was fused to previously described plasmids encoding hen egg lysozyme (HEL) targeted to the ER or the cytosol [34,35] (see methods). The ovalbumin-derived SIINFEKL peptide is presented by H2K^b ^and cell surface expression of K^b^-SIINFEKL complexes was assessed by staining with the 25-D1.16 monoclonal antibody [36]. As shown in Figure 1B, EL4 stably transfected clones, denoted EL4/HEL-ER-SIINFEKL and EL4/HEL-Cyto-SIINFEKL, can process and present SIINFEKL derived from an ER-localized or a cytosolic chimeric protein, respectively. These two clones, which display similar amounts of K^b^-SIINFEKL at the cell surface, were used in further experiments.

### UPR activation impairs MHC I surface expression

Various pharmacological agents are widely used to induce ER stress. For instance, tunicamycin and dithiothreitol are known to cause ER stress by preventing N-linked glycosylation or disrupting disulfide bond formation in the ER, respectively [37,38]. However, since MHC I proteins are glycosylated and contain disulfide bonds, we surmised that tunicamycin and dithiothreitol would directly hinder the assembly of MHC I molecules. We elected to use more physiological ER stress stimuli that should have less drastic effects on the synthesis of MHC I molecules: palmitate and glucose starvation. Palmitate is a saturated fatty acid recently shown to cause ER stress by disrupting mainly the structure and integrity of the ER [39-41]. Palmitate is abundant in the 'high fat Western diet', which renders this type of stress more physiological [42]. Glucose starvation is a common condition present for instance in vascularized bulky tumors and metastases, and is also a prototypical and strong inducer of ER stress [43].

**Figure 2:**
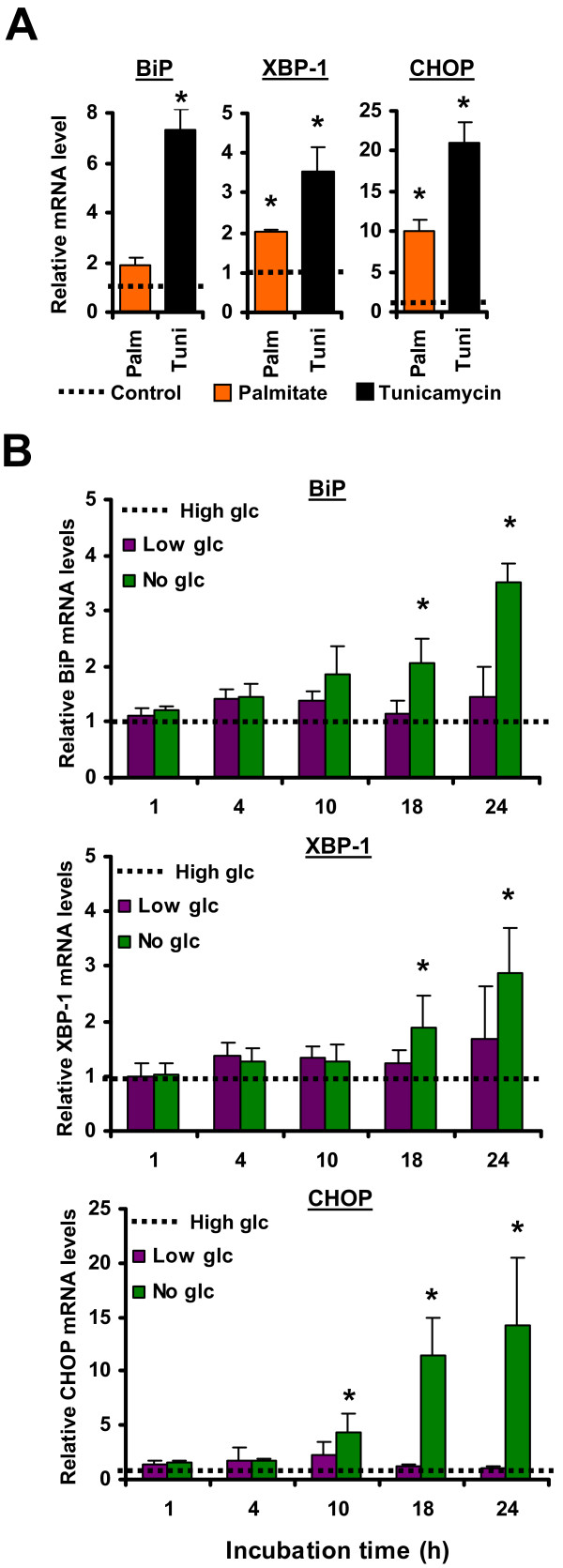
**Induction of ER stress in EL4 cells**. (A) UPR activation induced by palmitate treatment. EL4/HEL-Cyto-SIINFEKL cells were either non-treated or treated with 0.25 mM of palmitate or 2.5 μg/ml of tunicamycin for 18 hours. BiP, XBP-1 and CHOP mRNA levels were analyzed by RT-qPCR. Expression levels were normalized to the endogenous control gene β-actin. Transcript levels of treated cells were compared with basal mRNA values of untreated cells (*dotted line*), which were set to 1. (B) UPR activation induced by glucose deprivation. EL4 stable cell lines were incubated in DMEM medium lacking glucose or containing low glucose (1 mg/ml) or high glucose (4.5 mg/ml) for different durations. BiP, XBP-1 and CHOP mRNA levels were analyzed by RT-qPCR. Expression levels were normalized to the endogenous control gene β-actin. Transcript levels of cells incubated under low *(purple) *or no glucose *(green) *were compared to levels of cells grown in high glucose medium *(dotted line)*, which were set to 1. Similar results were obtained with EL4/HEL-ER-SIINFEKL cells (data not shown). Bars represent the mean and SD from three independent experiments performed in triplicate. *P < 0.05 when comparing untreated with palmitate- or tunicamycin-treated cells, or high glucose with low glucose or no glucose conditions.

Activation of the UPR was monitored by quantitative real-time reverse transcriptase polymerase chain reaction (RT-qPCR) analysis of BiP, CHOP and the normal and spliced XBP-1 transcripts, which are known to be induced during ER stress [19,44]. As expected, treatment of both EL4 transfectants, EL4/HEL-Cyto-SIINFEKL and EL4/HEL-ER-SIINFEKL (data not shown), with palmitate for 18 hours induced a mild UPR that was similar in both EL4 cell lines and of lesser magnitude than that induced by tunicamycin stimulation (Figure 2A). Similarly, we monitored UPR induction in EL4 cell lines grown in high glucose (4.5 mg/ml), low glucose (1 mg/ml) or no glucose-containing medium for different time durations (Figure 2B). BiP, XBP-1 and CHOP transcripts were significantly induced in both EL4 cell lines when they were completely deprived of glucose for 18 or 24 hours, indicating activation of the UPR under these conditions. However, none of these UPR markers were upregulated in cells grown in low glucose-containing medium, suggesting that 1 mg/ml of glucose is sufficient to keep the homeostasis of the ER in EL4 cells. The notable point here is that glucose starvation for 18–24 h induced a robust UPR that seemed to be of greater magnitude than that induced by palmitate (Figures 2A and 2B). Thus, ER stress induced by palmitate treatment or glucose starvation activates the UPR in EL4 cells, albeit to different extents.

**Figure 3:**
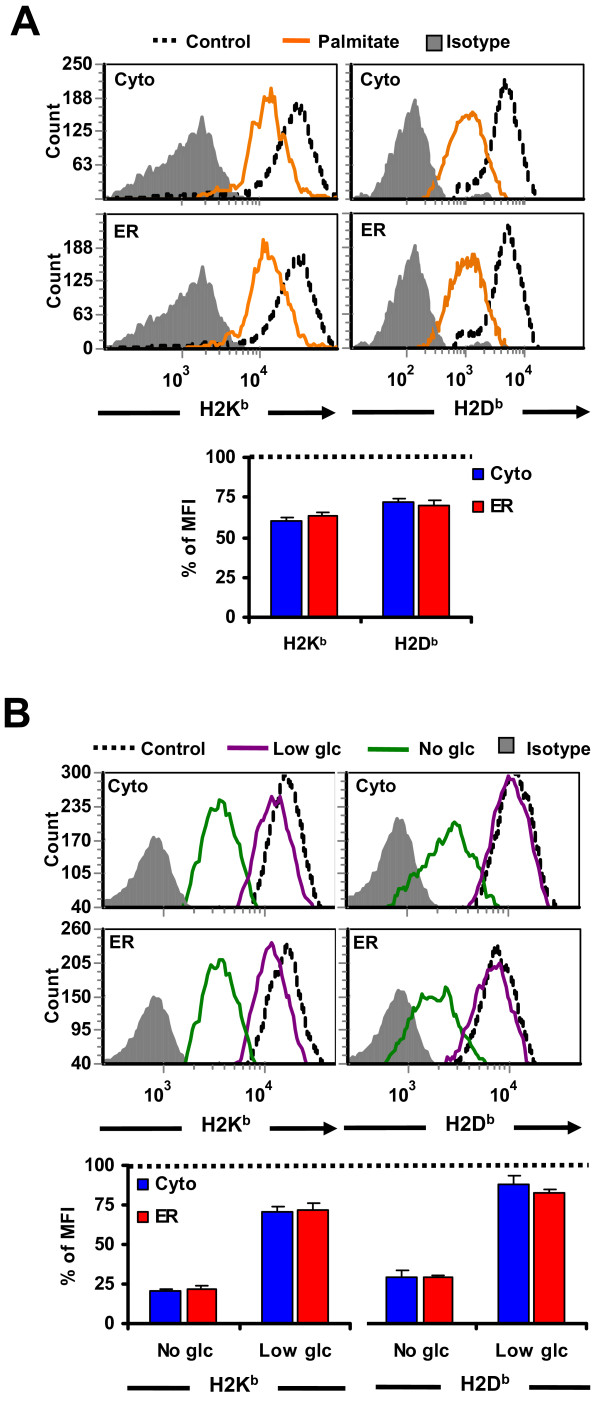
**ER stress impairs MHC I surface expression**. (A) Decreased MHC I surface expression induced by palmitate treatment. EL4 cells were either non-treated (*dotted line*) or treated with 0.25 mM of palmitate (*orange*) for 18 hours. EL4/HEL-Cyto-SIINFEKL (*top*) and EL4/HEL-ER-SIINFEKL *(bottom*) cells were stained with antibodies against H2K^b^, H2D^b ^or the corresponding isotypic control and analyzed by flow cytometry. Representative histograms of one of three independent experiments are depicted. Bars represent % of MFI intensity in treated EL4/HEL-Cyto-SIINFEKL (*blue*) and EL4/HEL-ER-SIINFEKL *(red*) cells relative to untreated cells (*dotted line*). Differences between untreated and treated cells are all significant (*P *< 0.05). (B) Decreased MHC I surface expression induced by glucose deprivation. EL4 cells were incubated in medium lacking glucose (*green*) or containing low glucose (1 mg/ml) (*purple*) or high glucose (4.5 mg/ml) (*dotted line*) for 18 hours and analyzed as in *A*. Bars represent % of MFI intensity in glucose-deprived EL4/HEL-Cyto-SIINFEKL (*blue*) and EL4/HEL-ER-SIINFEKL *(red*) cells relative to untreated cells (*dotted line*). Bars represent the mean and SD from three independent experiments performed in triplicate. Differences between control and glucose-deprived cells are all significant (*P *< 0.05).

To evaluate the effect of the UPR on MHC I expression, we quantified by flow cytometry surface levels of H2K^b ^and H2D^b ^in both EL4 cell lines submitted to ER stress (Figure 3). Cells in later apoptotic stages were excluded from the analysis by gating on propidium iodide-negative cells. Activation of the UPR with palmitate reduced cell surface expression of H2D^b ^and H2K^b ^by 30–40% in both cell lines (Figure 3A). Likewise, we evaluated whether UPR induced by glucose deprivation also affected MHC I surface expression. EL4 stable cell lines were incubated in medium lacking glucose or containing low glucose (1 mg/ml) or high glucose (4.5 mg/ml) for 18 hours and MHC I surface levels were measured by flow cytometry (Figure 3B). MHC I expression was impaired in cells grown both in low or no glucose conditions, albeit to a different extent. Cells that were completely deprived of glucose expressed only 25–30% of normal H2K^b ^and H2D^b ^levels, similar to the decline produced by tunicamycin (not shown). On the contrary, cells incubated in low glucose medium were less affected since around 70–90% of normal H2K^b ^and H2D^b ^levels were detected. Of note, a glucose dose of only 1 mg/ml was sufficient to raise MHC I levels by around three-fold (compare no-glucose with low glucose conditions in Figure 3B). As observed in the case of palmitate treatment, glucose starvation caused a similar downregulation of MHC I in the two stable cell lines (Figure 3B). These results show that ER stress induced by glucose deprivation or palmitate treatment causes decreased expression of surface MHC I molecules in EL4 cells.

### Posttranscriptional mechanism(s) cause decreased expression of surface MHC I molecules during ER stress

**Figure 4:**
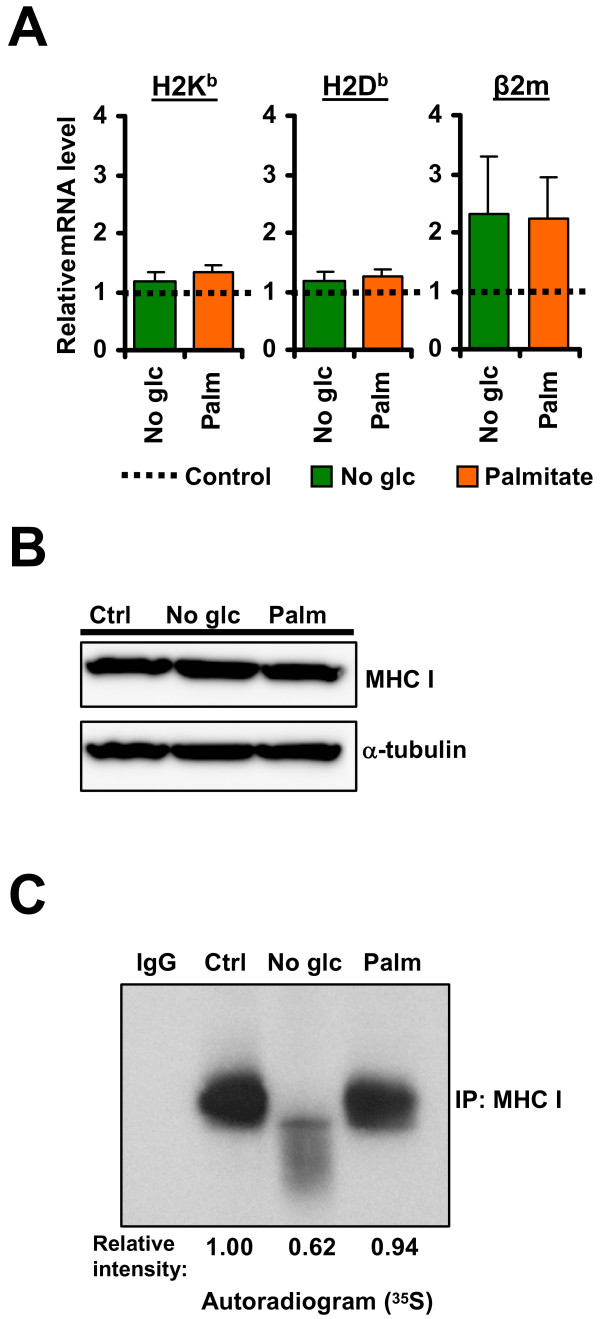
**ER stress impairs cell surface MHC I expression through posttranscriptional mechanism(s)**. EL4 cells were incubated in DMEM control medium containing glucose (4.5 mg/ml), or in medium lacking glucose or supplemented with 0.25 mM of palmitate for 18 hours. (A) ER stress does not decrease MHC I mRNA levels. H2K^b^, H2D^b ^and β2m mRNA levels were assessed and analyzed by RT-qPCR. Expression levels were normalized to the endogenous control gene β-actin. Transcript levels of glucose-starved (*green*) or palmitate-treated (*orange*) cells were compared with basal mRNA values of control cells (*dotted line*), which were set to 1. Bars represent the mean and SD from three independent experiments performed in triplicate. No significant differences were detected between untreated and treated cells (*P *< 0.05). (B) ER stress does not affect total MHC I protein amount. MHC I proteins from whole cell lysates were detected by Western blot with anti-MHC I antibodies. α-tubulin was used as loading control. A representative image of three independent experiments is shown. (C) ER stress differentially affects synthesis of MHC I. EL4 cells were incubated in control conditions, deprived of glucose or treated with 0.25 mM of palmitate for 17 hours and pulse-labeled with [^35^S]methionine/[^35^S]cysteine for 1 hour. Cell extracts were lysed and subjected to immunoprecipitation with anti-MHC I antibody or the corresponding isotypic antibody. Immunoprecipitated proteins were separated by SDS-PAGE and analyzed by fluorography. One representative experiment out of two is shown.

Since the UPR blocks transcription of numerous genes and can provoke premature degradation of mRNAs encoding secreted or membrane proteins [22], we investigated whether decreased MHC I surface expression was due to downregulation of MHC I transcripts. Using RT-qPCR, we found that mRNA expression levels of H2K^b^, H2D^b ^and β2m were unaffected in glucose-deprived or palmitate-treated cells (Figure 4A). In fact, the abundance of the β2m transcript, whose protein is essential for the formation of stable MHC I-peptide complexes, tended to increase in stressed cells relative to control cells (although this increase was not statistically significant). We therefore conclude that UPR induced with palmitate or glucose starvation leads to posttranscriptional attenuation of cell surface MHC I molecules.

To test whether diminished MHC I upon ER stress occurred only at the cell surface, we quantified total MHC I protein amount from whole lysates of cells previously treated with palmitate or deprived of glucose. We found that none of these conditions affected the steady state level of MHC I (Figure 4B). Nevertheless, one of the consequences of UPR activation is attenuation of protein synthesis [18]. Thus, we tested whether the UPR could impact on synthesis of MHC I in metabolically-labeled EL4 cells previously subjected to glucose deprivation or palmitate treatment for 18 hours. We found that glucose starvation, and to a much lesser extent palmitate, curtailed the synthesis of new MHC I molecules by around 40% and 5%, respectively (Figure 4C). Of note, the MHC I band in the no-glucose condition migrated faster than the bands in the control and the palmitate conditions. This effect is likely due to incomplete glycosylation of MHC I molecules in glucose-deprived cells. Thus, under our experimental conditions, ER stress did not affect the level of MHC I transcripts nor the total amount of MHC I protein (Figure 4A and 4B), but decreased the synthesis of new MHC I molecules (Figure 4C) and the amount of MHC I molecules at the cell surface (Figure 3).

### Decreased overall protein synthesis hinders MHC I-peptide presentation during ER stress

**Figure 5:**
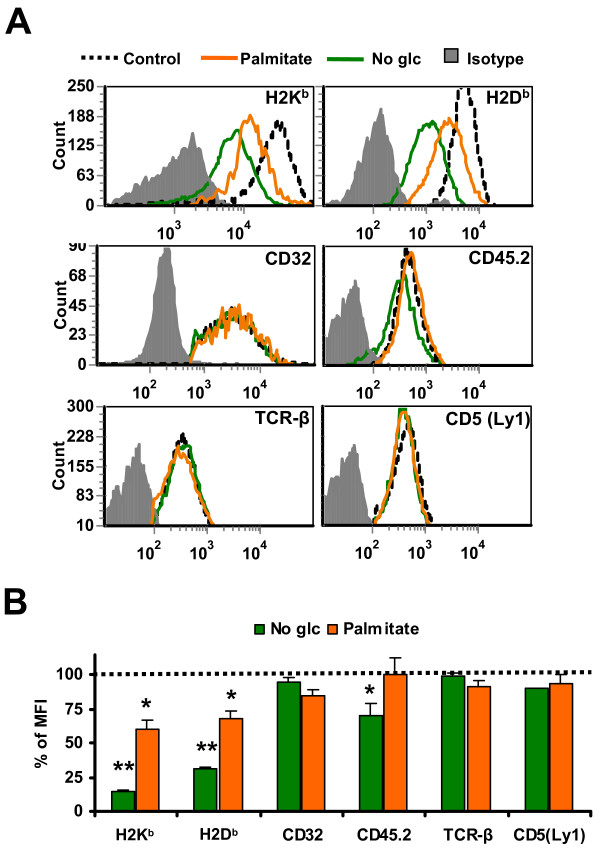
**Differential effects of ER stress on surface expression of various glycosylated proteins**. EL4 cells were incubated in high glucose (4.5 mg/ml) medium (*dotted line*) or in medium lacking glucose (*green*) or supplemented with 2.5 mM of palmitate (*orange*) for 18 hours. (A) Effect of glucose starvation or palmitate treatment on surface expression of glycosylated proteins. Surface expression of H2K^b^, H2D^b^, CD32, CD45.2, TCR-β and CD5 (Ly1) was determined by flow cytometry analysis. Representative histograms of one of three independent experiments are depicted. (B) Comparative effect of palmitate treatment and glucose starvation on surface expression of glycosylated proteins. Bars represent % of MFI intensity in glucose-starved (*green*) or palmitate-treated (*orange*) cells relative to control cells (*dotted line*). Bars represent the mean and SD from three independent experiments. *P < 0.05 and **P < 0.01 when comparing no glucose or palmitate with control conditions.

To understand how ER stress decreased cell surface expression of MHC I proteins, we evaluated the impact of ER stress on surface expression of a variety of glycoproteins (Figure 5). As shown before, palmitate treatment and glucose starvation severely impacted MHC I surface level. In contrast, surface expression of glycoproteins CD32, CD45.2, TCR-β and CD5 (Ly1) was minimally or not affected. These results show that the deleterious impact of the UPR is more severe on surface MHC I expression than on other glycoproteins. This suggests that reduction in the amount of cell surface MHC I molecules during ER stress cannot be attributed solely to defective MHC I synthesis. That contention is further supported by two elements. First, a 5% decline of MHC I synthesis in palmitate-treated cells (Figure 4C) is not commensurate with a 30–40% reduction of MHC I molecules at the cell surface (Figure 3A). Second, the total amount of MHC I proteins was not affected in stressed cells (Figure 4B), suggesting that MHC I proteins were relatively stable and that they did not reach the cell surface because they were not properly loaded with their peptide cargo and were therefore retained in the ER.

**Figure 6:**
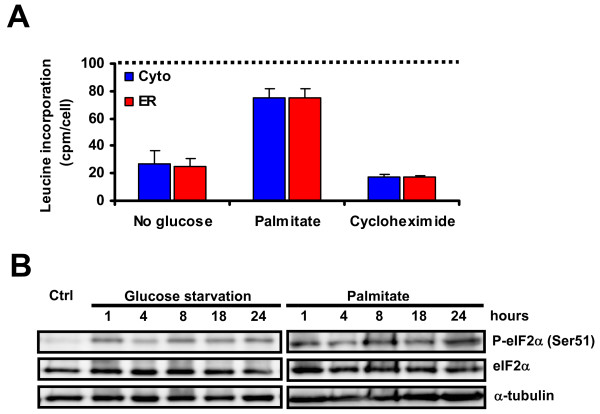
**ER stress inhibits protein synthesis through phosphorylation of eIF2α in EL4 stable cell lines**. (A) Decreased overall rate of protein synthesis upon ER stress. EL4 stable cell lines were deprived of glucose, treated with 0.25 mM of palmitate, 100 μg/ml of cycloheximide or cultured under control conditions for 19 hours. [^3^H]Leucine (10 μCi/mL) was added during the last hour. The rate of protein synthesis was measured by [^3^H]leucine incorporation. The results are expressed as the % of [^3^H]leucine incorporation per cell relative to control cells (*dotted line*) in EL4/HEL-Cyto-SIINFEKL (*blue*) and EL4/HEL-ER-SIINFEKL *(red*) cells. Bars depict the mean and SD of one representative experiment performed in triplicate. Differences between untreated and treated cells are all significant (*P *< 0.05). (B) Phosphorylation of eIF2α. EL4 cells were deprived of glucose or treated with 0.25 mM of palmitate for different durations over a 24-h period. Total cell lysates were immunoblotted against phosphorylated eIF2α (Ser51) or total eIF2α. α-tubulin was used as loading control. One representative experiment out of three is shown.

Assembly and presentation of MHC I-peptide complexes at the cell surface requires peptide delivery to the ER [28,45]. Since MHC I binding peptides derive mostly from recently synthesized proteins [23], we investigated whether glucose starvation and palmitate treatment attenuated protein translation. To test this idea we determined the rate of global protein synthesis in ER-stressed EL4 cells by measuring the rate of [^3^H]leucine incorporation. Translation was severely compromised in cells deprived of glucose, which showed a 75% decline in the rate of protein synthesis (Figure 6A). The impact on protein synthesis was comparable to that observed with the translation inhibitor cycloheximide (Figure 6A). Protein synthesis was less attenuated in palmitate-treated cells, but yet decreased by approximately 25%. Of note, ER stress produced similar inhibition of protein synthesis in EL4/HEL-Cyto-SIINFEKL and EL4/HEL-ER-SIINFEKL cells.

Following UPR signaling, inhibition of cap-dependent translation occurs via phosphorylation of Ser51 of the translation initiation factor eIF2α by activated PERK [18]. In line with this, we detected a rapid phosphorylation of eIF2α in EL4 cells after only 1 hour of glucose deprivation or treatment with palmitate (Figure 6B). This phosphorylated form persisted for 24 hours in both cases. These results show that eIF2α-mediated inhibition of protein synthesis occurs during glucose starvation or palmitate treatment and support the idea that impaired surface MHC I expression is caused by an inadequate peptide supply.

### Differential cell surface presentation of ER- vs. cytosol-derived peptide by MHC I molecules during ER stress

**Figure 7:**
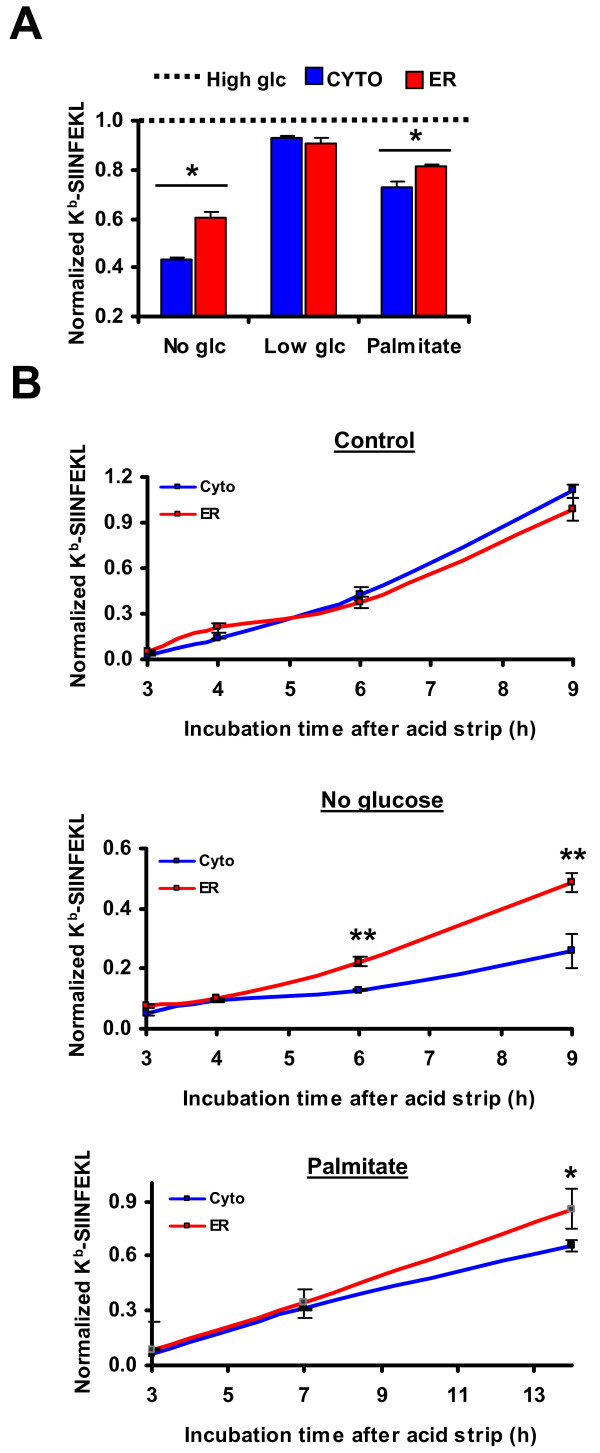
**Increased presentation of SIINFEKL peptide derived from ER-localized relative to cytosolic HEL protein during ER stress**. (A) ER stress differentially affects surface expression of K^b^-SIINFEKL complexes. EL4 stable cell lines were incubated in medium lacking glucose or containing low glucose (1 mg/ml) or high glucose (4.5 mg/ml) or supplemented with palmitate (2.5 mM) for 18 hours. K^b^-SIINFEKL abundance was assessed with the 25-D1.16 monoclonal antibody and APC-conjugated anti-mouse IgG_1 _antibody. Graph represents MFI values of glucose-deprived EL4/HEL-Cyto-SIINFEKL (*blue*) or EL4/HEL-ER-SIINFEKL (*red*) cells normalized to values of control cells, which were set to 1 (*dotted line*). (B) ER stress differentially affects surface expression of newly generated K^b^-SIINFEKL complexes. EL4 stable cell lines were incubated under control conditions (*top*), deprived of glucose (*middle*) or treated with 0.25 mM of palmitate (*bottom*) for 18 hours. Existent MHC-I complexes were eluted by acid strip and expression of new K^b^-SIINFEKL complexes was assessed as in *A *at the indicated times. MFI values of unstripped cells incubated under normal conditions and representing normal level of K^b^-SIINFEKL in each cell line were used to normalize MFI values of stripped cells. Bars represent the mean and SD from three independent experiments performed in triplicate. *P< 0.05 and **P < 0.01 when comparing normalized K^b^-SIINFEKL expression in EL4/HEL-Cyto-SIINFEKL with that of EL4/HEL-ER-SIINFEKL.

In the next series of experiments, we studied the impact of ER stress on MHC I-peptide presentation, using the SIINFEKL peptide as a model. K^b^-SIINFEKL surface expression was quantified by flow cytometry in EL4 stable cell lines submitted to ER stress by glucose deprivation or palmitate treatment for 18 hours. We found that abundance of cell surface K^b^-SIINFEKL decreased by more than 40% in cells that were completely deprived of glucose relative to control cells (Figure 7A). Similarly, K^b^-SIINFEKL complexes were diminished by 20% or more in the presence of palmitate and by 10% in cells grown in the presence of low glucose. Thus, consistent with what we observed in the case of surface MHC I molecules, MHC I-peptide presentation is reduced during ER stress.

In addition, we found that although K^b^-SIINFEKL expression was reduced in both cell lines upon ER stress, EL4/HEL-ER-SIINFEKL cells presented significantly more complexes than EL4/HEL-Cyto-SIINFEKL cells (Figure 7A). This difference occurred during complete glucose starvation, or treatment with palmitate (Figure 7A) or tunicamycin (not shown), but not when the glucose concentration was low, suggesting that it is UPR-specific (Figure 7A). Of note, both cell lines displayed similar amounts of K^b^-SIINFEKL complexes under normal conditions (Figure 1B and Figure 7B top). We wish to emphasize that differences in abundance of K^b^-SIINFEKL among the two types of EL4 transfectants during ER stress (Figure 7A) cannot be ascribed to an overall difference in expression of H2K^b ^at the cell surface (Figure 3A and 3B). We therefore conclude that during ER stress, diminution of K^b^-SIINFEKL presentation was more drastic when the peptide derived from a protein localized in the cytosol than from an ER-retained protein.

Cell surface K^b^-SIINFEKL complexes have been shown to be very stable [46]. We therefore postulated that monitoring K^b^-SIINFEKL in the aforementioned experimental conditions might lead us to underestimate the impact of ER stress on exportation of "new" MHC I-peptide complexes at the cell surface. Thus, in the next series of experiments, we took advantage of the fact that cell surface MHC I-peptide complexes can be disrupted by mild acid elution at pH 3.3 [47-49]. EL4 stable cell lines were submitted or not to ER stress, then existent K^b^-SIINFEKL complexes were acid stripped and generation of new complexes was measured at different time points. We reasoned that in this way we could directly assess the effect of the UPR on the generation of new K^b^-SIINFEKL complexes. In control conditions, cells rapidly re-expressed K^b^-SIINFEKL and initial control levels were reached 9 hours after acid stripping (Figure 7B, top). Notably, EL4/HEL-ER-SIINFEKL and EL4/HEL-Cyto-SIINFEKL cell lines showed similar kinetics. In contrast, stressed cells were not able to reach basal amount of K^b^-SIINFEKL after acid strip (Figure 7B, middle and bottom). This effect was more striking in glucose-starved than in palmitate-treated cells, consistent to what we observed for MHC I expression (Figure 5B). Remarkably, EL4/HEL-ER-SIINFEKL cells generated significantly more cell surface K^b^-SIINFEKL complexes than EL4/HEL-Cyto-SIINFEKL during ER stress (Figure 7B, middle and bottom). It should be noted that it was not possible to measure generation of complexes at time points later than 9 hours after acid strip, since at this time cells had already been stressed for 24 hours and cell death became a confounding variable. We conclude that ER stress decreases presentation of both existent (Figure 7A) and newly generated (Figure 7B) K^b^-SIINFEKL complexes and that it differentially affected abundance of SIINFEKL derived from an ER- vs. a cytosol-localized protein.

### Changes in stability of cytosolic and ER-retained HEL during ER stress

**Figure 8:**
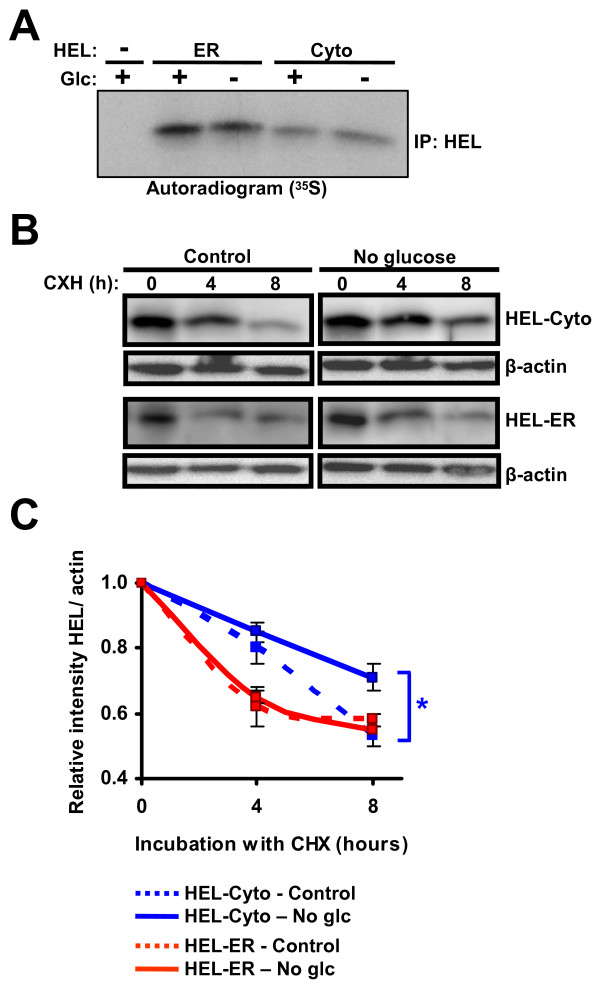
**Stability of cytosolic HEL and ER-retained HEL during ER stress**. (A) Rate of synthesis of cytosolic HEL and ER-retained HEL. EL4/HEL-ER-SIINFEKL and EL4/HEL-Cyto-SIINFEKL cells were incubated in control conditions (4.5 mg/ml) or deprived of glucose for 17 hours and pulse-labeled with [^35^S]methionine/[^35^S]cysteine for 1 hour. Cell extracts were lysed and subjected to immunoprecipitation with anti-HEL antibody. Immunoprecipitated proteins were separated by SDS-PAGE and analyzed by fluorography. One representative experiment out of two is shown. (B) Stability of cytosolic HEL and ER-retained HEL. EL4/HEL-ER-SIINFEKL and EL4/HEL-Cyto-SIINFEKL cell lines were incubated in control conditions (4.5 mg/ml) or deprived of glucose for 18 hours. Then, 100 μg/ml of cycloheximide were added to inhibit protein synthesis and cell lysates taken at different times were immunoblotted against HEL or β-actin (used as loading control). One representative immunoblot out of three is shown. (C) Graph represents relative intensities of HEL (means and SD) from three independent experiments. *P < 0.05 when comparing control vs. no glucose conditions.

As mentioned above, newly synthesized proteins are the major substrates for MHC I processing. In addition, it has been shown that the protein synthesis machinery of the cytosol and ER compartments is under distinct regulatory control during the UPR [50]. Thus the differential effect of ER stress on presentation of ER- or cytosol-derived SIINFEKL could be due to changes in the translation rates of the source proteins. We explored this possibility and compared the synthesis rate of HEL-ER and HEL-Cyto in EL4 stable cell lines under normal conditions and during glucose starvation in metabolic labeling experiments. The rate of synthesis of cytosolic HEL and ER-retained HEL was not affected by glucose deprivation (Figure 8A). Hence, the different abundance of SIINFEKL at the surface of these cell lines during ER stress is not due to changes in the rate of synthesis of the precursor proteins.

MHC I-peptide presentation not only relies on protein synthesis but also on protein degradation. Therefore, we explored whether the stability of these proteins could be differentially affected during ER stress. EL4 stable cell lines were deprived or not of glucose for 18 hours and then treated with cycloheximide to inhibit protein synthesis. The protein levels of cytosolic HEL and ER-retained HEL were assessed by Western blot thereafter. We observed an increased stability of cytosolic HEL in the absence of glucose compared to control conditions (Figure 8B and 8C). In contrast, the stability of ER-retained HEL was the same in control conditions and during glucose starvation. These results suggest that reduced presentation of SIINFEKL by H2K^b ^when the peptide derives from the cytosolic protein compared to the ER-retained protein is due to increased stability of the cytosolic protein during ER stress.

## Discussion

The ER stands at the crossroad of two fundamental cellular processes: MHC I antigen presentation and UPR activation during ER stress. The UPR regulates protein synthesis and degradation, chaperoning and decay of ER mRNAs [14,15]. Thus, it has enormous potential to impinge on MHC I antigen processing which relies on all these processes. Here, we assessed the effect of ER stress on the final outcome of antigen processing and presentation: MHC I-peptide abundance. We demonstrated that ER stress induced by tunicamycin, palmitate or glucose deprivation, decreases peptide presentation by MHC I molecules. This finding is consistent with prior studies reporting reduced MHC I surface levels in human cells expressing a mutant HFE protein or overexpressing transcriptionally active isoforms of UPR-activated transcription factors ATF-6 and XBP-1 [51,52]. Recently, reduced expression of MHC I molecules was also observed in antigen presenting cells during palmitate treatment [53]. Thus, diminution of MHC I surface expression upon UPR activation appears to be a generalized phenomenon occurring during ER stress induced by a variety of stimuli (pharmacological agents, mutant proteins, glucose starvation and saturated fatty acid).

Since the UPR provokes the degradation of ER-localized mRNAs [22], accelerated decay of MHC I mRNA might have been responsible for the reduction of cell surface MHC I expression. However, the presence of normal levels of MHC I and β2m transcripts allowed us to exclude this possibility. During ER stress, transducers of the UPR seek to decrease the ER burden by suppressing translation initiation through phosphorylation of eIF2α by activated PERK [14,15]. We demonstrated that inhibition of protein synthesis and phosphorylation of eIF2α did occur in EL4 cells treated with palmitate or deprived of glucose. Of note, the effect of these two treatments on phosphorylation of eIF2α was similar, yet inhibition of overall protein synthesis was more severe in glucose-deprived than in palmitate-treated cells. We presume that this discrepancy was due to brisk inhibition of the mammalian target of rapamycin (mTOR) pathway during glucose starvation [54]. Inhibition of mTOR blocks phosphorylation of p70 ribosomal S6 kinase and eukaryotic initiation factor 4E binding protein 1 and thereby leads to inhibition of protein synthesis. Given the dramatic inhibition of protein synthesis during glucose starvation, it was notable that the translation rate of the two HEL variants was not affected. That feature of our HEL variants is not unique as there are several proteins whose synthesis is unaffected during ER stress [9,54].

We found that ER stress-induced inhibition of overall protein synthesis curtails the synthesis of new MHC I molecules. Nevertheless, we do not believe that decreased synthesis of MHC I proteins per se was a leading factor responsible for decreased levels of MHC I molecules at the cell surface. Our assertion is based on three lines of evidence: i) a 5% decline of MHC I synthesis in palmitate-treated cells was not commensurate with a drop of 30–40% of surface MHC I, ii) during ER stress, cell surface levels of MHC I proteins were decreased much more than those of other glycoproteins that must also pass through the same maturation process and quality control in order to be exported at the cell surface, and iii) the total amount of intracellular MHC I proteins was not decreased during stress suggesting that MHC I molecules did not reach the cell surface mainly because they were sequestered in the ER. In addition, de Almeida et al. showed that a partial UPR signaling induced by overexpression of ATF-6 or XBP-1 in the absence of genuine stress stimulus also resulted in decreased MHC I surface expression [52]. MHC I heavy chains and β2m are present in excess in the ER. The limiting factor in the assembly and presentation of MHC I-peptide complexes is peptide delivery to the ER [28,45]. Moreover, peptides presented by MHC I molecules derive mainly from proteins that are degraded a few seconds or minutes after their synthesis as opposed to stable proteins with a slow turnover. Thus, generation of MHC I peptide ligands is tightly coupled to ongoing protein synthesis and inhibition of translation rapidly decreases the amount of cell surface MHC I-peptide complexes [55]. Our favorite hypothesis is therefore that decreased MHC I presentation during ER stress is due mainly, albeit not exclusively, to restriction of peptide availability. Given that MHC I molecules preferentially sample polypeptides that are being actively translated [55], we posit that global attenuation of protein synthesis caused by palmitate and glucose starvation limits the amount of a vast repertoire of peptides available for insertion in MHC I molecules. Nevertheless, we do not exclude the possibility that defective synthesis of MHC I and other possible mechanisms such as inappropriate loading of peptides, contribute to diminution of MHC I-peptide presentation. This would be mainly the case of peptides deriving from proteins whose synthesis is not curtailed upon ER stress. For instance, our results show that ER stress diminished presentation of K^b^-SIINFEKL complexes even though the synthesis of the proteins source of this particular peptide (HEL variants) was not affected.

A main conclusion of our work is that ER stress-induced attenuation of MHC I-peptide presentation is more severe when the source protein is localized in the cytosol than in the ER. The difference between proteins in these two cell compartments was UPR-specific because it did not occur in the low glucose condition in which no UPR markers were significantly induced. Our cell lines expressing HEL-Cyto-SIINFEKL and HEL-ER-SIINFEKL displayed identical responses to palmitate treatment or glucose starvation. The two cell lines showed similar upregulation of UPR markers and equivalent reduction in cell surface levels of H2K^b ^and H2D^b ^during ER stress. Despite the fact that the translation rates and degradation profiles normally differ in both cell lines, they displayed similar levels of K^b^-SIINFEKL complexes under steady-state conditions. On the contrary, presentation of K^b^-SIINFEKL complexes was differentially affected in these cell lines during ER stress. Only 1–2 out of every 10,000 peptides generated by the proteasome bind to MHC I molecules [28]. Our data therefore beg the question: how would an ER-retained protein generate more peptides than a cytosolic protein during ER stress? We showed that this difference was not due to variations in the translation rate of each precursor protein during ER stress. This suggests that differences in peptide presentation resulted from discrepancies in the degradation of ER vs. cytosolic proteins during ER stress. UPR transducers specifically enhance degradation of proteins in the secretory pathway in order to decrease the ER folding load. During ER stress, cotranslational protein translocation is inhibited and newly-synthesized ER proteins are triaged for degradation (ERAD) [38,56,57]. Furthermore, retrotranslocation of ER-resident proteins in the cytosol for proteasomal degradation is enhanced [58]. Based on this, we expected to see an increased degradation of the ER-retained HEL variant during ER stress. However, the stability of the ER-retained protein remained unchanged while the stability of the cytosolic HEL variant increased during ER stress. The most parsimonious explanation for the latter findings would be that during ER stress, proteasomes focus primarily on degradation of ER as opposed to cytosolic proteins. This would be consistent with the fact that the primary role of the UPR is to decrease the folding burden in the stressed ER. We therefore propose that regulation of proteasomal degradation during ER stress leads to a reduction in MHC I peptide ligands generated from cytosolic precursors. Further studies will be needed to determine whether this concept can be generalized to other proteins and other MHC I-associated peptides.

What might be the impact of the UPR on immune recognition of infected and neoplastic cells? Paradoxically, if the decreased generation of MHC I-peptide complexes results mainly from inhibition of translation, it could facilitate recognition of virus-infected cells. Phosphorylation of eIF2α hampers canonical cap-dependent translation initiation which regulates synthesis of 95–98% of cellular mRNAs [9]. However, some viruses can use internal ribosomal entry sites in their 5' noncoding region to initiate cap-independent translation [9,59]. Thus, by preferentially repressing presentation of self peptides, the UPR could facilitate recognition of viral peptides (the needle in the haystack [60]). The potential impact of the UPR on recognition of neoplastic cells is not inherently obvious. On the one hand, by repressing production of MHC I-peptide complexes, the UPR may hinder presentation of tumor antigens to CD8 T cells. Indeed, generation of optimal CD8 T cell responses is promoted by high epitope density on antigen presenting cells [61,62]. However, an elegant study by Schwab *et al. *has shown that upon induction of eIF2α phosphorylation by ER stress, cells can generate MHC I-associated peptides derived from cryptic translational reading frames [63]. Expression of such cryptic peptides by neoplastic cells might trigger recognition of stressed cells by CD8 T lymphocytes. Finally, a high fat diet rich in saturated fatty acids such as palmitate, could potentiate the conditions of ER stress found in tumour cells and lessen even more MHC I-peptide presentation. In fact, obesity has been associated with increased susceptibility to infection and impaired immune responses [53,64]. We anticipate that high-throughput sequencing of MHC I-associated peptides [33] will be necessary to comprehensively evaluate how ER stress molds the peptide repertoire (in terms of both abundance and diversity), and to gain further insights into the global impact of the UPR on recognition of stressed cells by CD8 T lymphocytes.

## Conclusion

Our work shows that ER stress impinges on the MHC I peptide repertoire in two ways: by decreasing overall MHC I-peptide presentation and by changing the relative contribution of ER- vs. cytosol-proteins to the MHC I peptide repertoire. Since ER stress is a characteristic feature of infection and malignancy, dysregulation of MHC I-peptide presentation could have major implications in the recognition of infected and transformed cells by CD8 T lymphocytes.

## Methods

### Cell lines

EL4 cells were maintained in Dulbecco's modified Eagle's medium (DMEM) (GIBCO Burlington, ON, Canada) supplemented with 5% fetal bovine serum (FBS) and antibiotics. EL4 stable transfectants were grown in the same medium supplemented with 1000 μg/ml of G418.

### DNA constructs

pHYK/HEL-ER/myc and pCMV/HEL-Cyto/myc plasmids encoding ER-retained or cytoplasmic HEL, respectively, were provided by S. Ostrand-Rosenberg (University of Maryland, Baltimore, USA). The pHYR/HEL-ER plasmid contains the HEL gene (that includes a signal sequence) fused to the ER-retention signal KDEL, whereas pCMV/HEL-Cyto codes for HEL with a modified N-terminus and lacks ER-retention signal. These plasmids have successfully been shown to target HEL to the ER or to the cytosol [34,35]. pHYK/HEL-ER and pCMV/HEL-Cyto were sequenced to ascertain correct sequence and reading frame. Fragments coding for HEL-ER or HEL-Cyto were fused by PCR to the region coding for the ovalbumin-derived peptide SIINFEKL, flanked by a sequence of 18 bp (LEQLE-SIINFEKL-TEWTS, here referred to as SIINFEKL) to ensure proteasome- and TAP-dependent peptide processing [65,66]. PCR amplification products were subcloned into the pPCR-Script Amp cloning vector (Stratagene, Cedar Creek, TX, USA). HEL-ER-SIINFEKL or HEL-Cyto-SIINFEKL were excised and cloned into the bicistronic pIRES-EGFP2 vector (Clontech, Mountain View, CA, USA) to generate pIRES-EGFP2/HEL-ER-SIINFEKL and pIRES-EGFP2/HEL-Cyto-SIINFEKL (Figure 1A). Both constructs were sequenced to ascertain correct sequence and reading frame.

### Stable transfectants

EL4/HEL-ER-SIINFEKL and EL4/HEL-Cyto-SIINFEKL were generated by transfecting EL4 cells with the appropriate HEL-containing pIRES-EGFP2 plasmid. Transfections were done with Lipofectamine LTX Reagent (Invitrogen, Burlington, ON, Canada) as instructed by the manufacturer. 24 hours after transfection, single cells expressing the brightest signal of EGFP were sorted by fluorescence-activated cell sorting (FACS) on a FACSAria cell sorter (BD Biosciences, Mississauga, ON, Canada). Stable transfected clones were further selected by drug resistance (1000 μg/ml of G418) in combination with repeated cycles of FACS of EGFP-positive cells. Clones expressing similar levels of K^b^-SIINFEKL at the cells surface were selected for use in further experiments.

### Stress induction

ER stress was induced by incubating cells in fresh medium containing 0.25 mM of palmitate or 2.5 μg/ml of tunicamycin (Sigma-Aldrich, St. Louis, MO, USA) for the indicated times. Palmitate was prepared as described previously [67] and delivered as a complex with fatty acid-free BSA. Glucose starvation was induced by culturing cells in glucose and sodium pyruvate-free or in low glucose (1000 mg/L) DMEM medium (GIBCO) supplemented with 5% dialyzed FBS and antibiotics for the indicated times. Control cells were grown in high glucose DMEM medium, containing 4500 mg/L of glucose and 110 mg/L of sodium pyruvate supplemented with 5% FBS and antibiotics.

### Flow cytometry

MHC I molecules at the cell surface were stained with biotin-conjugated anti-H2K^b ^(clone AF6-88.5) and biotin-conjugated anti-H2D^b ^(clone KH95), followed by PeCy5 or APC-conjugated streptavidin. Other cell surface glycosylated proteins were stained with FITC-conjugated anti-CD45.2, FITC-conjugated anti-CD5 (Ly1), APC-conjugated anti-TCR-β and PE-conjugated anti-CD32. All antibodies were purchased from BD Biosciences. K^b^-SIINFEKL levels were determined with the 25-D1.16 antibody [36] followed by staining with APC-conjugated anti-mouse IgG_1 _(Clone X56). Propidium iodide (BD Biosciences) was used to exclude cells in later apoptotic stages from the analysis. Cells were analyzed on a BD LSR II flow cytometer using FACSDiva (BS Biosciences) and FCS Express softwares (De Novo Software, Los Angeles, CA, USA) [68,69].

### Acid strip assay

MHC I-peptide complexes were eluted with acid treatment as previously described [47-49]. Briefly, cells (~5 × 10^5^) were resuspended in 0.2 ml of citrate phosphate buffer at pH 3.3 (0.131 M citric acid/0.066 M Na_2_HPO_4_, NaCl 150 mM) for 1 minute, neutralized with appropriate medium pH 7.4 and either reincubated in fresh medium or stained for flow cytometry analysis.

### RNA extraction, reverse transcription and RT-qPCR

Total RNA was isolated with TRIzol reagent (Invitrogen) according to the manufacturer's instructions. Purified RNA was reverse transcribed using the High Capacity cDNA reverse transcription Kit with random primers (Applied Biosystems, Foster City, CA, USA) as described by the manufacturer. A reference RNA (Stratagene, La Jolla, CA, USA) was also transcribed in cDNA. Expression level of target genes was determined using primer and probe sets from Universal ProbeLibrary  or Applied Biosystems (ABI Gene Expression Assays or SYBR green PCR Master Mix, ). Primer sequences are given in Additional file 1. RT-qPCR assay for XBP-1 was designed to amplify both the normal and spliced forms. Pre-developed TaqMan^® ^assays for β-actin were used as endogenous controls. RT-qPCR analyses were performed as described using a PRISM^® ^7900 HT Sequence Detection System (Applied Biosystems) [70]. The relative quantification of target genes was determined by using the ΔΔCT (cycle threshold) method. Relative expression (RQ) was calculated using the Sequence Detection System (SDS) 2.2.2 software (Applied Biosystems) and the formula RQ = 2^-ΔΔCT^.

### Protein synthesis and metabolic labeling

To measure protein synthesis, EL4 cell lines were cultured in presence or absence of glucose (4.5 mg/ml) for 18 hours. [^3^H]Leucine (10 μCi/mL) was added during the last hour. Cells were washed twice with ice-cold PBS and fixed for 30 minutes on ice with 10% TCA. Cells were then rinsed with water and lysed with 0.1 N NaOH. Radioactivity incorporation was determined with a liquid scintillation analyzer Tri-CArb 2800TR (Perkin Elmer).

In vivo biosynthetic labeling experiments were carried out as described previously [71]. Briefly, to evaluate the rate of synthesis of HEL and MHC I, EL4 cell lines were grown in control conditions or in the presence of 2.5 mM of palmitate or in the absence of glucose for 17 hours. After this period, 10^7 ^cells per condition were starved of methionine and cysteine for 30 min. ^35^S-labeled methionine and cysteine (220 μCi/mL) were then added for 1 hour. Cells were harvested and lysed in Triton X-100 buffer (50 mM Tris pH7.5, 150 mM NaCl, 1% Triton X-100, 1 mM EDTA, 40 mM β-glycerophosphate) supplemented with complete protease inhibitor cocktail (Roche Molecular Biochemicals, Laval, QC, Canada) and phosphatase inhibitors (1 mM Na_3_VO_4 _and 5 mM NaF). Immunoprecipitation of ER-retained HEL or cytosolic HEL and MHC I were performed using anti-HEL antibody purchased from Affinity BioReagents (Golden, CO, USA) or anti-H2K^b ^or anti-H2D^b ^hybridoma culture supernatans antibody [49], according to the method described previously [71]. Proteins were separated by SDS-PAGE and labeled proteins were detected by fluorography.

### Immunoblotting

EL4 cell lines were cultured under control conditions or submitted to glucose deprivation or palmitate treatment (0.25 mM) for the indicated times. When indicated, 100 μg/mL of cycloheximide (Sigma-Aldrich) was used for various durations to measure the stability of HEL variants. Cells were harvested and lysed in Triton X-100 buffer. The lysates were cleared by centrifugation and the protein content was measured by the Bradford method (Biorad, Mississauga, ON, Canada). Samples were resolved by SDS-PAGE and immunoblotted with the following antibodies: anti-β-actin (AC-15) from Sigma-Aldrich, anti-HEL from Affinity BioReagents, anti-MHC class I (2G5) from Santa Cruz Biotechnology Inc. (Santa Cruz, CA, USA), anti-α-tubulin, anti-phospho-eIF2α (Ser51), anti-eIF2α and horseradish peroxidase (HRP)-conjugated anti-rabbit IgG from Cell Signaling Technology (Beverly, MA, USA), and HRP-conjugated goat anti-mouse IgG from BD Pharmigen (San Diego, CA, USA). Chemiluminescent signal was detected using a LAS3000 imaging system (Fujifilm, Tokyo, Japan) and quantification of band intensities was done using the Multi Gauge v3.0 (Fujifilm) and the ImageQuaNT v5.0 (Molecular Dynamics, Sunnyvale, CA, USA) softwares.

### Statistical analysis

The means of normally distributed data were compared using the Student *t *test, with a *P *value of < 0.05 considered significant. Data are presented as the mean and SD. Whenever the results are expressed as a percentage of control, the statistical analysis was performed on the actual value.

## Abbreviations

APC: antigen presenting cell; β2m: β_2_-microglobulin; ER: endoplasmic reticulum; ERAD: ER-associated degradation; FACS: fluorescence-activated cell sorting; HEL: hen egg lysozyme; MHC I: major histocompatibility complex class I; mTOR: mammalian target of rapamycin; RT-qPCR: quantitative real-time reverse transcriptase polymerase chain reaction; UPR: unfolded protein response.

## Authors' contributions

DPG designed the study, carried out experiments and analyzed the data. PLT designed and carried out metabolic labeling experiments. MPH and DDV participated in flow cytometry experiments. EC participated in molecular cloning experiments. SM designed biochemical experiments. CP conceived and designed the study. DPG and CP drafted the manuscript, and all authors edited and approved the final manuscript.

## References

[B1] Wong P, Pamer EG (2003). CD8 T cell responses to infectious pathogens. Annu Rev Immunol.

[B2] Zitvogel L, Tesniere A, Kroemer G (2006). Cancer despite immunosurveillance: immunoselection and immunosubversion. Nat Rev Immunol.

[B3] Shin BK, Wang H, Yim AM, Le Naour F, Brichory F, Jang JH, Zhao R, Puravs E, Tra J, Michael CW (2003). Global profiling of the cell surface proteome of cancer cells uncovers an abundance of proteins with chaperone function. J Biol Chem.

[B4] Gleimer M, Parham P (2003). Stress management: MHC class I and class I-like molecules as reporters of cellular stress. Immunity.

[B5] Marciniak SJ, Ron D (2006). Endoplasmic reticulum stress signaling in disease. Physiol Rev.

[B6] Wiemann K, Mittrucker HW, Feger U, Welte SA, Yokoyama WM, Spies T, Rammensee HG, Steinle A (2005). Systemic NKG2D down-regulation impairs NK and CD8 T cell responses in vivo. J Immunol.

[B7] Raulet DH (2003). Roles of the NKG2D immunoreceptor and its ligands. Nat Rev Immunol.

[B8] Hickman-Miller HD, Hildebrand WH (2004). The immune response under stress: the role of HSP-derived peptides. Trends Immunol.

[B9] Holcik M, Sonenberg N (2005). Translational control in stress and apoptosis. Nat Rev Mol Cell Biol.

[B10] Mamane Y, Petroulakis E, LeBacquer O, Sonenberg N (2006). mTOR, translation initiation and cancer. Oncogene.

[B11] Bi M, Naczki C, Koritzinsky M, Fels D, Blais J, Hu N, Harding H, Novoa I, Varia M, Raleigh J (2005). ER stress-regulated translation increases tolerance to extreme hypoxia and promotes tumor growth. EMBO J.

[B12] Moenner M, Pluquet O, Bouchecareilh M, Chevet E (2007). Integrated endoplasmic reticulum stress responses in cancer. Cancer Res.

[B13] Carrasco DR, Sukhdeo K, Protopopova M, Sinha R, Enos M, Carrasco DE, Zheng M, Mani M, Henderson J, Pinkus GS (2007). The differentiation and stress response factor XBP-1 drives multiple myeloma pathogenesis. Cancer Cell.

[B14] Schroder M, Kaufman RJ (2005). The mammalian unfolded protein response. Annu Rev Biochem.

[B15] Ron D, Walter P (2007). Signal integration in the endoplasmic reticulum unfolded protein response. Nat Rev Mol Cell Biol.

[B16] Rutkowski DT, Arnold SM, Miller CN, Wu J, Li J, Gunnison KM, Mori K, Sadighi Akha AA, Raden D, Kaufman RJ (2006). Adaptation to ER stress is mediated by differential stabilities of pro-survival and pro-apoptotic mRNAs and proteins. PLoS Biol.

[B17] Szegezdi E, Logue SE, Gorman AM, Samali A (2006). Mediators of endoplasmic reticulum stress-induced apoptosis. EMBO Rep.

[B18] Harding HP, Zhang Y, Bertolotti A, Zeng H, Ron D (2000). Perk is essential for translational regulation and cell survival during the unfolded protein response. Mol Cell.

[B19] Yoshida H, Matsui T, Yamamoto A, Okada T, Mori K (2001). XBP1 mRNA is induced by ATF6 and spliced by IRE1 in response to ER stress to produce a highly active transcription factor. Cell.

[B20] Yoshida H, Matsui T, Hosokawa N, Kaufman RJ, Nagata K, Mori K (2003). A time-dependent phase shift in the mammalian unfolded protein response. Dev Cell.

[B21] Lee AH, Iwakoshi NN, Glimcher LH (2003). XBP-1 regulates a subset of endoplasmic reticulum resident chaperone genes in the unfolded protein response. Mol Cell Biol.

[B22] Hollien J, Weissman JS (2006). Decay of endoplasmic reticulum-localized mRNAs during the unfolded protein response. Science.

[B23] Yewdell JW, Nicchitta CV (2006). The DRiP hypothesis decennial: support, controversy, refinement and extension. Trends Immunol.

[B24] Eisenlohr LC, Huang L, Golovina TN (2007). Rethinking peptide supply to MHC class I molecules. Nat Rev Immunol.

[B25] Rammensee HG, Falk K, Rotzschke O (1993). Peptides naturally presented by MHC class I molecules. Annu Rev Immunol.

[B26] Heemels MT, Ploegh H (1995). Generation, translocation, and presentation of MHC class I-restricted peptides. Annu Rev Biochem.

[B27] Pamer E, Cresswell P (1998). Mechanisms of MHC class I-restricted antigen processing. Annu Rev Immunol.

[B28] Yewdell JW, Reits E, Neefjes J (2003). Making sense of mass destruction: quantitating MHC class I antigen presentation. Nat Rev Immunol.

[B29] Shastri N, Cardinaud S, Schwab SR, Serwold T, Kunisawa J (2005). All the peptides that fit: the beginning, the middle, and the end of the MHC class I antigen-processing pathway. Immunol Rev.

[B30] Golovina TN, Wherry EJ, Bullock TN, Eisenlohr LC (2002). Efficient and qualitatively distinct MHC class I-restricted presentation of antigen targeted to the endoplasmic reticulum. J Immunol.

[B31] Leifert JA, Rodriguez-Carreno MP, Rodriguez F, Whitton JL (2004). Targeting plasmid-encoded proteins to the antigen presentation pathways. Immunol Rev.

[B32] Caron E, Charbonneau R, Huppe G, Brochu S, Perreault C (2005). The structure and location of SIMP/STT3B account for its prominent imprint on the MHC I immunopeptidome. Int Immunol.

[B33] Fortier MH, Caron E, Hardy MP, Voisin G, Lemieux S, Perreault C, Thibault P (2008). The MHC class I peptide repertoire is molded by the transcriptome. J Exp Med.

[B34] Armstrong TD, Clements VK, Martin BK, Ting JP, Ostrand-Rosenberg S (1997). Major histocompatibility complex class II-transfected tumor cells present endogenous antigen and are potent inducers of tumor-specific immunity. Proc Natl Acad Sci USA.

[B35] Qi L, Rojas JM, Ostrand-Rosenberg S (2000). Tumor cells present MHC class II-restricted nuclear and mitochondrial antigens and are the predominant antigen presenting cells in vivo. J Immunol.

[B36] Porgador A, Yewdell JW, Deng Y, Bennink JR, Germain RN (1997). Localization, quantitation, and in situ detection of specific peptide-MHC class I complexes using a monoclonal antibody. Immunity.

[B37] Murray JI, Whitfield ML, Trinklein ND, Myers RM, Brown PO, Botstein D (2004). Diverse and specific gene expression responses to stresses in cultured human cells. Mol Biol Cell.

[B38] Kang SW, Rane NS, Kim SJ, Garrison JL, Taunton J, Hegde RS (2006). Substrate-specific translocational attenuation during ER stress defines a pre-emptive quality control pathway. Cell.

[B39] Jeffrey KD, Alejandro EU, Luciani DS, Kalynyak TB, Hu X, Li H, Lin Y, Townsend RR, Polonsky KS, Johnson JD (2008). Carboxypeptidase E mediates palmitate-induced beta-cell ER stress and apoptosis. Proc Natl Acad Sci USA.

[B40] Guo W, Wong S, Xie W, Lei T, Luo Z (2007). Palmitate modulates intracellular signaling, induces endoplasmic reticulum stress, and causes apoptosis in mouse 3T3-L1 and rat primary preadipocytes. Am J Physiol Endocrinol Metab.

[B41] Borradaile NM, Han X, Harp JD, Gale SE, Ory DS, Schaffer JE (2006). Disruption of endoplasmic reticulum structure and integrity in lipotoxic cell death. J Lipid Res.

[B42] Bray GA, Popkin BM (1998). Dietary fat intake does affect obesity!. Am J Clin Nutr.

[B43] Ozcan U, Ozcan L, Yilmaz E, Duvel K, Sahin M, Manning BD, Hotamisligil GS (2008). Loss of the tuberous sclerosis complex tumor suppressors triggers the unfolded protein response to regulate insulin signaling and apoptosis. Mol Cell.

[B44] Lee AS (2005). The ER chaperone and signaling regulator GRP78/BiP as a monitor of endoplasmic reticulum stress. Methods.

[B45] Neefjes JJ, Momburg F, Hammerling GJ (1993). Selective and ATP-dependent translocation of peptides by the MHC-encoded transporter. Science.

[B46] Kukutsch NA, Rossner S, Austyn JM, Schuler G, Lutz MB (2000). Formation and kinetics of MHC class I-ovalbumin peptide complexes on immature and mature murine dendritic cells. J Invest Dermatol.

[B47] Sugawara S, Abo T, Kumagai K (1987). A simple method to eliminate the antigenicity of surface class I MHC molecules from the membrane of viable cells by acid treatment at pH 3. J Immunol Methods.

[B48] Storkus WJ, HJ Zeh, Salter RD, Lotze MT (1993). Identification of T-cell epitopes: rapid isolation of class I-presented peptides from viable cells by mild acid elution. J Immunother Emphasis Tumor Immunol.

[B49] Perreault C, Jutras J, Roy DC, Filep JG, Brochu S (1996). Identification of an immunodominant mouse minor histocompatibility antigen (MiHA). T cell response to a single dominant MiHA causes graft-versus-host disease. J Clin Invest.

[B50] Stephens SB, Dodd RD, Brewer JW, Lager PJ, Keene JD, Nicchitta CV (2005). Stable ribosome binding to the endoplasmic reticulum enables compartment-specific regulation of mRNA translation. Mol Biol Cell.

[B51] de Almeida SF, Carvalho IF, Cardoso CS, Cordeiro JV, Azevedo JE, Neefjes J, de Sousa M (2005). HFE cross-talks with the MHC class I antigen presentation pathway. Blood.

[B52] de Almeida SF, Fleming JV, Azevedo JE, Carmo-Fonseca M, de Sousa M (2007). Stimulation of an unfolded protein response impairs MHC class I expression. J Immunol.

[B53] Shaikh SR, Mitchell D, Carroll E, Li M, Schneck J, Edidin M (2008). Differential effects of a saturated and a monounsaturated fatty acid on MHC class I antigen presentation. Scand J Immunol.

[B54] Hay N, Sonenberg N (2004). Upstream and downstream of mTOR. Genes Dev.

[B55] Qian SB, Reits E, Neefjes J, Deslich JM, Bennink JR, Yewdell JW (2006). Tight linkage between translation and MHC class I peptide ligand generation implies specialized antigen processing for defective ribosomal products. J Immunol.

[B56] Oyadomari S, Yun C, Fisher EA, Kreglinger N, Kreibich G, Oyadomari M, Harding HP, Goodman AG, Harant H, Garrison JL (2006). Cotranslocational degradation protects the stressed endoplasmic reticulum from protein overload. Cell.

[B57] Shenkman M, Tolchinsky S, Lederkremer GZ (2007). ER stress induces alternative nonproteasomal degradation of ER proteins but not of cytosolic ones. Cell Stress Chaperones.

[B58] Oda Y, Okada T, Yoshida H, Kaufman RJ, Nagata K, Mori K (2006). Derlin-2 and Derlin-3 are regulated by the mammalian unfolded protein response and are required for ER-associated degradation. J Cell Biol.

[B59] Tardif KD, Mori K, Siddiqui A (2002). Hepatitis C virus subgenomic replicons induce endoplasmic reticulum stress activating an intracellular signaling pathway. J Virol.

[B60] Yewdell JW (2007). Plumbing the sources of endogenous MHC class I peptide ligands. Curr Opin Immunol.

[B61] Wherry EJ, Puorro KA, Porgador A, Eisenlohr LC (1999). The induction of virus-specific CTL as a function of increasing epitope expression: responses rise steadily until excessively high levels of epitope are attained. J Immunol.

[B62] Henrickson SE, Mempel TR, Mazo IB, Liu B, Artyomov MN, Zheng H, Peixoto A, Flynn MP, Senman B, Junt T (2008). T cell sensing of antigen dose governs interactive behavior with dendritic cells and sets a threshold for T cell activation. Nat Immunol.

[B63] Schwab SR, Shugart JA, Horng T, Malarkannan S, Shastri N (2004). Unanticipated antigens: translation initiation at CUG with leucine. PLoS Biol.

[B64] Falagas ME, Kompoti M (2006). Obesity and infection. Lancet Infect Dis.

[B65] Nussbaum AK, Dick TP, Keilholz W, Schirle M, Stevanovic S, Dietz K, Heinemeyer W, Groll M, Wolf DH, Huber R (1998). Cleavage motifs of the yeast 20S proteasome beta subunits deduced from digests of enolase 1. Proc Natl Acad Sci USA.

[B66] Craiu A, Akopian T, Goldberg A, Rock KL (1997). Two distinct proteolytic processes in the generation of a major histocompatibility complex class I-presented peptide. Proc Natl Acad Sci USA.

[B67] Diakogiannaki E, Morgan NG (2008). Differential regulation of the ER stress response by long-chain fatty acids in the pancreatic beta-cell. Biochem Soc Trans.

[B68] Meunier MC, Delisle JS, Bergeron J, Rineau V, Baron C, Perreault C (2005). T cells targeted against a single minor histocompatibility antigen can cure solid tumors. Nat Med.

[B69] Blais ME, Brochu S, Giroux M, Belanger MP, Dulude G, Sekaly RP, Perreault C (2008). Why T cells of thymic versus extrathymic origin are functionally different. J Immunol.

[B70] Baron C, Somogyi R, Greller LD, Rineau V, Wilkinson P, Cho CR, Cameron MJ, Kelvin DJ, Chagnon P, Roy DC (2007). Prediction of graft-versus-host disease in humans by donor gene-expression profiling. PLoS Med.

[B71] Servant MJ, Coulombe P, Turgeon B, Meloche S (2000). Differential regulation of p27(Kip1) expression by mitogenic and hypertrophic factors: Involvement of transcriptional and posttranscriptional mechanisms. J Cell Biol.

